# Global Patterns in Seasonal Activity of Influenza A/H3N2, A/H1N1, and B from 1997 to 2005: Viral Coexistence and Latitudinal Gradients

**DOI:** 10.1371/journal.pone.0001296

**Published:** 2007-12-12

**Authors:** Brian S. Finkelman, Cécile Viboud, Katia Koelle, Matthew J. Ferrari, Nita Bharti, Bryan T. Grenfell

**Affiliations:** 1 Center for Infectious Disease Dynamics, Department of Biology, Eberly College of Science, The Pennsylvania State University, University Park, Pennsylvania, United States of America; 2 Fogarty International Center, National Institutes of Health, Bethesda, Maryland, United States of America; 3 Department of Ecology and Evolutionary Biology, University of Michigan, Ann Arbor, Michigan, United States of America; University of Cape Town, South Africa

## Abstract

Despite a mass of research on the epidemiology of seasonal influenza, overall patterns of infection have not been fully described on broad geographic scales and for specific types and subtypes of the influenza virus. Here we provide a descriptive analysis of laboratory-confirmed influenza surveillance data by type and subtype (A/H3N2, A/H1N1, and B) for 19 temperate countries in the Northern and Southern hemispheres from 1997 to 2005, compiled from a public database maintained by WHO (FluNet). Key findings include patterns of large scale co-occurrence of influenza type A and B, interhemispheric synchrony for subtype A/H3N2, and latitudinal gradients in epidemic timing for type A. These findings highlight the need for more countries to conduct year-round viral surveillance and report reliable incidence data at the type and subtype level, especially in the Tropics.

## Introduction

Influenza is a major human pathogen, the epidemiology of which is characterized by epidemics that occur seasonally throughout the world every year, with occasional pandemics arising from novel subtypes of the virus; both annual and pandemic influenza are the source of considerable morbidity, mortality, and economic burden [1]. In temperate regions, annual influenza epidemics typically occur during the winter months, both in the Northern Hemisphere (November through March) and in the Southern Hemisphere (April through September); in the Tropics, influenza activity can occur year-round with larger epidemics in between those found in the Northern and Southern Hemispheres [1]–[3]. Several direct and/or indirect environmental factors are thought to drive the seasonality of influenza—including indoor crowding during cold and wet seasons [4], increased virus survival in cold and dry conditions [5], and decreased immunity of the host, perhaps mediated by a decrease in Vitamin D synthesis from lack of sunlight during winter months [6]. However, the exact mechanism behind seasonality in influenza remains a topic of considerable controversy.

Two main types (A and B) of the influenza virus contribute to the disease burden in humans. Influenza A is generally more prevalent and leads to greater mortality in humans than influenza B, which is a significant source of morbidity but not mortality [7]. Additionally, influenza A is further classified into major subtypes based on genetic and antigenic differences in the membrane glycoproteins hemagglutinin and neuraminidase [7]. Currently, there are two major subtypes in circulation among humans, A/H3N2 (H3) and A/H1N1 (H1), with H3 accounting for more influenza-related mortality [1]. Antigenic evolution in influenza A is punctuated rather than continuous, characterized by the emergence of clusters of antigenically similar but genetically unique strains that dominate subtype incidence [8]. Additionally, although they carry distinct surface antigens, H3 and H1 have been shown to provide some level of cross-immunity to each other [9], [10]. As a result, interference competition for susceptible hosts may occur between the two subtypes, such that one subtype could potentially influence the dynamics of the other [11].

Traditionally, epidemiological studies of influenza incidence have relied on excess mortality data, such as the U.S. pneumonia and influenza death rates maintained by the Centers for Disease Control and Prevention (CDC) in Atlanta, because these data sets generally contained information on the best available spatial and temporal scales [1], [7], [12]. However, such data are not laboratory-confirmed; they measure severe disease burden rather than morbidity or disease transmission, and they do not discriminate between influenza types and subtypes. Although extremely useful, such data may miss important aspects of influenza population dynamics that can only be understood by investigating the similarities and differences between types and subtypes and their potential interactions.

Influenza specimens can now be easily and accurately typed or subtyped as H3, H1, or B using a number of methods, such as immunofluorescence assays for rapid antigen detection, virus culture and subsequent antigenic analysis, and reverse transcription polymerase chain reaction assays [13]. Furthermore, since the late 1990s, the World Health Organization (WHO) has maintained a record of weekly laboratory-confirmed cases of flu by type and subtype from a growing number of countries and has made these data available to the public through the online database FluNet [http://gamapserver.who.int/GlobalAtlas/home.asp]. Thus, it is now becoming possible to analyze influenza type and subtype incidence data on meaningful spatial and temporal scales.

Here we take advantage of the growing availability of viral surveillance data to provide a descriptive analysis of influenza type and subtype seasonal dynamics in 19 countries from temperate regions in both the Northern and Southern Hemispheres during the 1997 through 2005 influenza seasons. The analysis allowed us to address a number of open questions in annual influenza epidemiology [2], [3], [7]: 1) What overall patterns emerge from the type and subtype incidence data, and do these patterns support what is already commonly believed about influenza type and subtype population dynamics? 2) Do particular types or subtypes dominate seasonal epidemics at both the hemisphere and global level, and, if so, to what degree? 3) Are there any cyclical patterns of type or subtype incidence? and, 4) Are there any simple patterns governing the spatiotemporal spread of seasonal epidemics across broad geographic scales and are these patterns similar for different types or subtypes?

## Materials and Methods

Data were obtained from FluNet [http://gamapserver.who.int/GlobalAtlas/home.asp], an online service provided by the WHO. Data from all of the approximately 60 countries available on FluNet were analyzed initially, and from these, a subset of 19 countries with sufficient data over the available time period to allow for meaningful analyses were selected for further study. General parameters for each country (Table 1)—such as latitude, longitude, population size, area, and GDP—were obtained using the CIA World Factbook online database [https://www.cia.gov/library/publications/the-world-factbook/index.html]. These countries were all from temperate regions, and of the 19 countries, only Australia and Japan had truly year-round surveillance over the entire nine years studied, with the rest of the data limited to winter-centered seasons of variable length.

**Table 1:** Overall country comparisons of geography, demographics, and influenza virus surveillance data by type and subtype (A/H3N2, A/H1N1, and B).

Country	Latitude	Longitude	Population in millions*	Area (×1,000 sq km)	GDP (billion US$)*	Influenza A/H3 total no.†	Influenza A/H1 total no.†	Influenza B total no.†
Argentina	34°S	64°W	40.3	2,767	599.1	3,153	1,102	678
Chile	30°S	71°W	16.2	757	203	3,387	552	693
South Africa	29°S	24°E	44.0	1,220	576.4	859	488	417
Australia	27°S	133°E	20.4	7,687	666.3	2,128	324	726
Israel	31.5°N	34.75°E	6.4	21	166.3	1,314	345	409
Japan	36°N	138°E	127.4	378	4,220	27,299	11,097	15,143
United States	38°N	97°W	301.1	9,827	12,980	95,727	11,554	24,634
Portugal	39.5°N	8°W	10.6	92	203.1	1,442	206	536
Spain	40°N	4°W	40.4	505	1070	871	385	591
Italy	42.83°N	12.83°E	58.1	301	1,727	1,972	426	661
France	46°N	2°E	63.7	547	1,871	7,914	992	4,354
Romania	46°N	25°E	22.3	238	197.3	838	207	356
Switzerland	47°N	8°E	7.6	41	252.9	1,214	176	539
Germany	51°N	9°E	82.4	357	2585	11,040	3,561	2,847
United Kingdom	54°N	2°W	60.8	245	1,903	4,496	833	1,645
Denmark	56°N	10°E	5.5	43	198.5	329	53	171
Latvia	57°N	25°E	2.3	65	35.08	2,309	332	896
Norway	62°N	10°E	4.6	324	207.3	1,977	492	1,259
Finland	64°N	26°E	5.2	338	171.7	3,372	628	653

* Population and GDP values are most recent available figures, not averages during the study period.

† Subtype totals use adjusted data values and all types and subtypes are summed over the nine year study period.

Because influenza epidemics typically occur during winter months, a “season” in the Northern Hemisphere was defined as occurring from week 27 of one calendar year to week 26 of the following calendar year, so that one defined “season” would include an entire influenza epidemic (similar to [14]); a Southern Hemisphere “season” was defined as being the same as the calendar year. Furthermore, all influenza seasons, regardless of hemisphere, were labeled according to the calendar year in which they begin; for example, the 1997 influenza season in the U.S. was defined as being the epidemic that occurred during the winter from late 1997 through early 1998.

Raw data were available in the form of weekly numbers of isolates for each type and subtype; however, type A isolates in some instances were not further subtyped into H3 or H1. Thus, H3 and H1 weekly totals needed to be adjusted to reflect the presence of unsubtyped A isolates before being analyzed further. This adjustment was performed using the formula 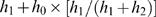, where *h_1_* is the number of weekly isolates for a given subtype (either H3 or H1), *h_0_* is the number of weekly isolates for type A that was not further subtyped, and *h_2_* is the number of weekly isolates for the other subtype. Of the total 212,636 influenza A isolates recorded across all study countries, 49% were not further subtyped.

To account for any variations in sample effort when making year to year comparisons, the relative size of an epidemic for a given type and subtype in a given country and year was determined as a percent of the total number of isolates in a given year belonging to a particular type and subtype. The relative contribution of different types and subtypes to the influenza activity within a given country could then be determined by comparing the mean percent values of these types and subtypes. The significance of differences in mean percent values was determined by Student's *t*-test.

A type or subtype was considered “dominant” in a given country if it accounted for 70% or more of the total influenza isolates for a particular season [12]. A type or subtype was considered “codominant” if it accounted for between 40% and 70% of the annual isolates. Additionally, hemispheres were considered to show “temporal overlap” in influenza activity for a particular type or subtype if at least one isolate of that type or subtype was recorded in both hemispheres during a given week.

Spatial synchrony between hemispheres was analyzed by finding the cross-correlation between the mean annual growth rates (*G*), defined as the geometric mean of the *G* values for the individual countries in the hemisphere, where *G* equals the annual sum of the weekly adjusted incidence data for one year divided by the annual sum for the previous year [15]. The annual growth rate reflects changes in type and subtype incidence and, thus, was good for comparisons of interannual population dynamics. To avoid any concerns regarding potential division by zero, the values for the weekly adjusted incidence data were all increased by one [16]. Periodicity in mean hemisphere growth rates was determined through autocorrelation analysis [16].

To examine spatiotemporal patterns in type and subtype epidemics, the mean week of the epidemic was used as a measure of epidemic timing. The mean week was calculated as a weighted average over the nine years studied, using the formula 
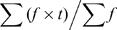
, where *f* is the adjusted number of weekly isolates and *t* is the week (1 through 52, starting from the first week of the season as previously defined) of the measurement, and the standard error was calculated for each country. This epidemic mean week value was correlated with the latitude of the approximate geographic center of the country of interest (Table 1). Latitude was also correlated with epidemic onset and duration. For computational purposes, an epidemic was defined as starting and ending at the first and last weeks in the season in which three consecutive weeks of nonzero numbers of isolates were obtained, respectively (adapted from [12]), respectively. Upon review, this definition seemed to produce reasonable results for epidemic onset in almost all cases; the lone exception was the 2000 H1 season in Finland, in which a small number of isolates appeared earlier than the main epidemic, and in this case the second week to meet the previously stated criterion was chosen instead. Finally, the significance of all correlations was determined using Student's *t*-test, where the test statistic is equal to 
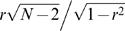
, where *r* is the correlation coefficient and *N* is the number of samples (in this case, 19 countries).

All analyses were performed using MATLAB Student 7.1, while Movies S1, S2 and S3 were created using R 2.4.0.

## Results

### Patterns of Seasonality and Dominance

**Figure 1:**
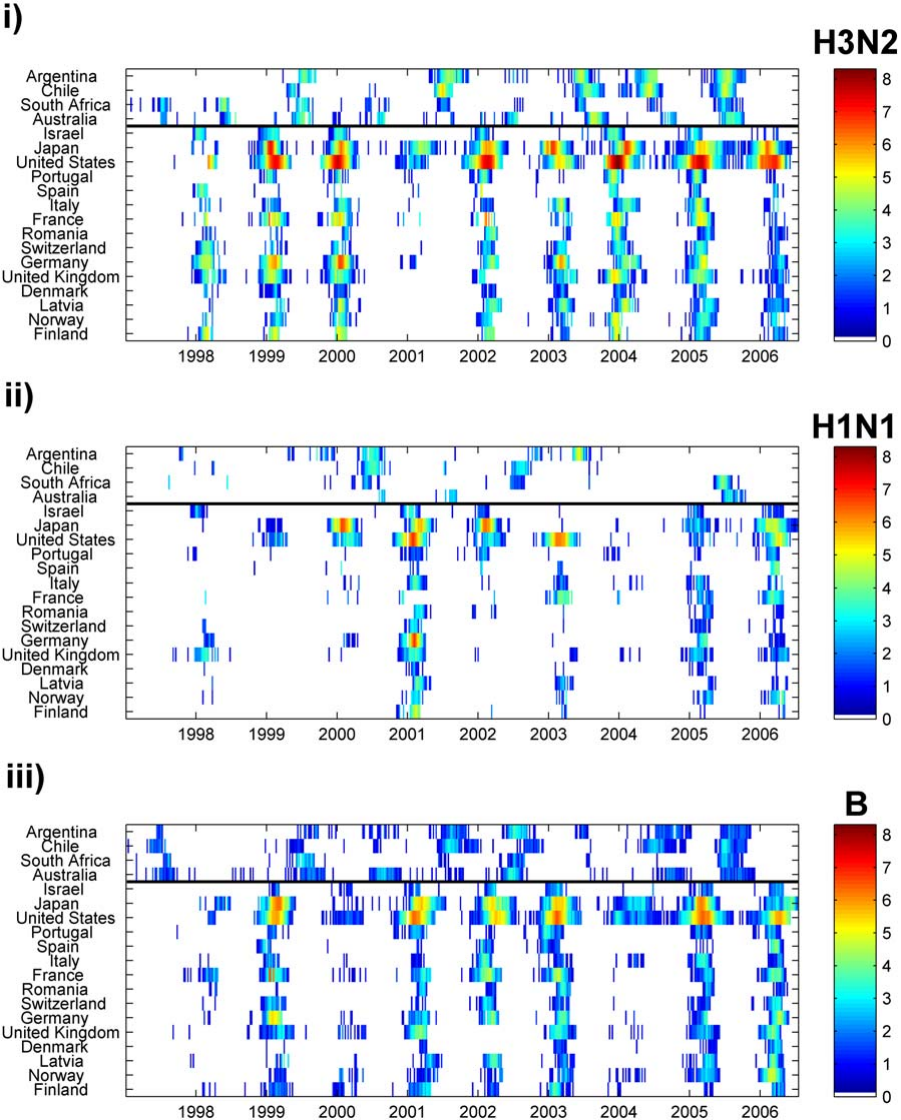
Summary of incidence data. Data on weekly number of isolates by type and subtype were collected from FluNet (WHO) and summarized for 19 countries—arranged from southernmost to northernmost, with the black line dividing the two hemispheres—for influenza seasons from 1997 to 2005. For H3 and H1 subtypes, totals were adjusted to account for type A isolates that were not further subtyped. For all types and subtypes, the adjusted number of weekly isolates was increased by one to remove any zero values. The natural log of the results were plotted by country for (i) H3, (ii) H1, and (iii) B on a color scale, with white representing either zero isolates or no data and brown representing the highest observed number of isolates for H3 (n = 4,057).

**Figure 2:**
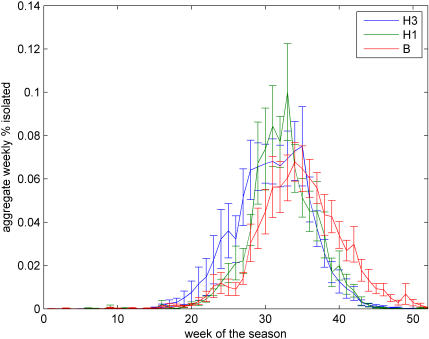
Mean aggregate incidence curves by type and subtype for the Northern Hemisphere. Weekly incidence values for each type and subtype were summed for the Northern Hemisphere for each of the nine study seasons and normalized as a percentage of the total number of isolates of that particular type or subtype recorded over the nine year study period. The average value for each week (±SE) were plotted versus time for each type and subtype. The profiles clearly show that influenza B lags the other two subtypes (see text).

All countries studied were located in temperate areas of the Northern and Southern Hemispheres, with latitudes ranging between 67°N and 34°S (Table 1). For H3, H1 and B and in all countries, epidemics were primarily confined to the winter months (Figure 1) (as seen in [1], [2]). However, considerable H3 and B activity was observed outside of the standard influenza season, with temporal overlap of influenza activity occurring between the two hemispheres on average 28±6% (SE) of the year for H3, 8±2% of the year for H1, and 32±8% of the year for B (Figure 1). Additionally, in the Northern Hemisphere, B epidemics were found to occur significantly later (mean epidemic week = week of 12 February) in the season on average than H3 or H1 epidemics (mean epidemic week = week of 16 and 27 January, respectively) (Tukey test, *p*<0.01) (Figure 2).

**Figure 3:**
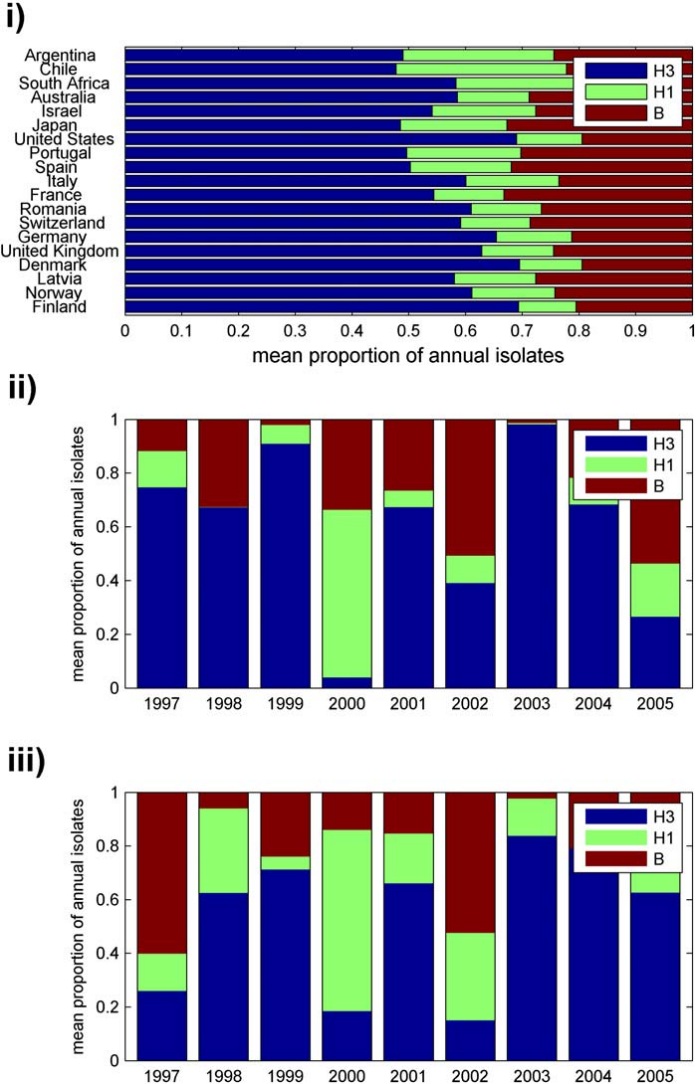
Type and subtype dominance. (i) Mean proportion of annual isolates belonging to each type or subtype, averaged across the nine study seasons, were plotted for each country. (ii–iii) The proportion of annual isolates belonging to each type or subtype was averaged across all countries in both the (ii) Northern and (iii) Southern hemisphere and plotted for each of the nine seasons.

In both hemispheres, H3 was more prevalent than either H1 or B, with a mean annual incidence significantly larger for H3 than H1 in 84% of countries and significantly larger for H3 than B in 63% of countries (*p*<0.05) (Figure 3i). There was no significant difference in mean annual incidence between H1 and B in any country. Additionally, H3 was dominant or codominant in most seasons, followed by B and H1 (Figure 3ii, 3iii) (as observed in [17]). On average, H3 was dominant or codominant in a given country in 6.2±0.3 (SE) of the 9 study seasons, H1 in 1.3±0.3 seasons, and B in 2.4±0.3 seasons. H1 and H3 were both codominant in the same season only once out of 171 total influenza seasons (9 seasons in each of the 19 countries), while B was codominant with either H3 or H1 in 19 seasons (Fisher's exact test, *p*<0.001). Furthermore, H3 epidemics were typically the most widespread geographically. On average, H3 was dominant or codominant in 68±8% (SE) of countries, H1 in 15±5% countries, and B in 27±5% of countries. The same type and subtype was dominant in both hemispheres in 7 of the 9 seasons; however, nondominant types and subtypes were often not present in similar proportions in both hemispheres in a given year (Figure 3). Even though they were not dominant as often as H3, H1 and B were always found somewhere in a given season, although there appeared to be no simple pattern of where they were found.

Additional information on the spatial-temporal patterns of influenza incidence is given in Movies S1, S2 and S3 while Figure S1 provides a summary of annual incidence by country and type and subtype.

### Type and Subtype Synchrony

**Figure 4:**
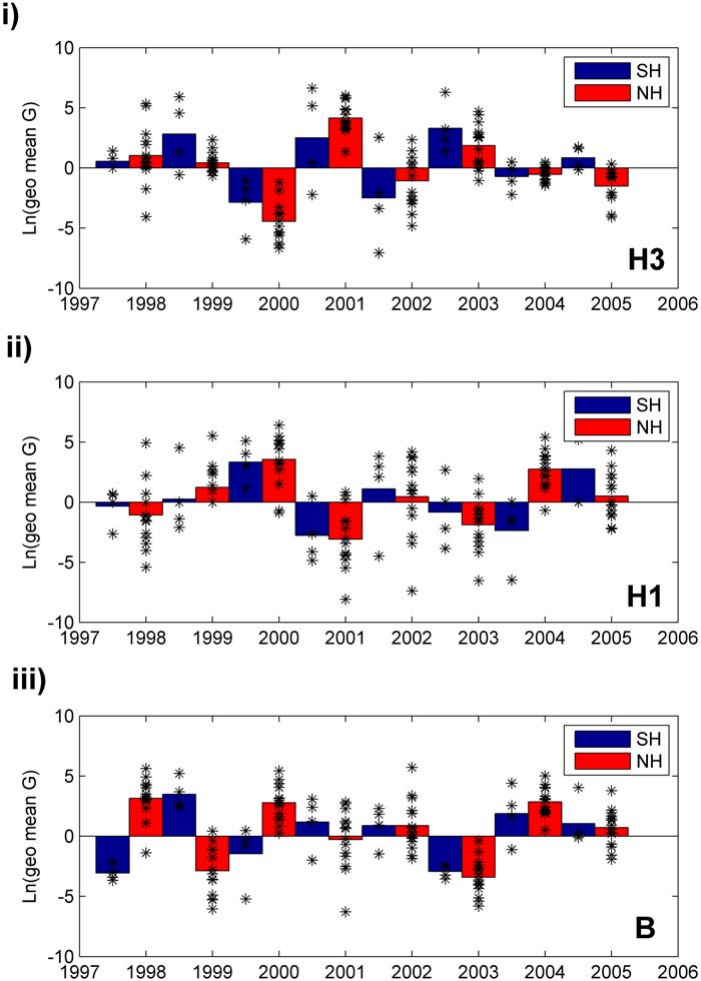
Annual type and subtype epidemic growth rates by hemisphere. The epidemic growth rate (*G*) was calculated for each country by dividing the annual sum of adjusted weekly isolates for year “t” by the sum for year “t-1.” The average value of *G* for each hemisphere was determined by taking the geometric mean of the individual country values. To emphasize the difference between positive and negative growth, the natural log of the growth rates were plotted for (i) H3, (ii) H1, and (iii) B. The scatter plot shows the values of *G* for individual countries in the given season, to give an idea of the observed variation. Note that the values for 1998 and 1999 in the Southern Hemisphere are based on only three countries, as Argentina had no data available during the 1998 influenza season.

To analyze type and subtype synchrony across countries and type and subtype periodicity within countries, we computed annual growth rates (*G*) for each type and subtype in each country between consecutive years and then calculated the cross-correlation between the mean values for each hemisphere (Materials and Methods). The two hemispheres showed evidence for synchrony in influenza dynamics in the case of H3 (correlation coefficient, r = 0.78, *p*<0.05); however, interhemispheric synchrony was not observed for either H1 (r = 0.51, *p*>0.05) or B (r = −0.18, *p*>0.05) (Figure 4) (comparable to [17]). Furthermore, there seemed to be no preference for one hemisphere to lead the other in changes in type or subtype incidence. Similar results were obtained when this analysis was performed using type and subtype proportions instead of raw numbers of positive isolates (results not shown).

### Patterns in Spatiotemporal Spread

**Figure 5:**
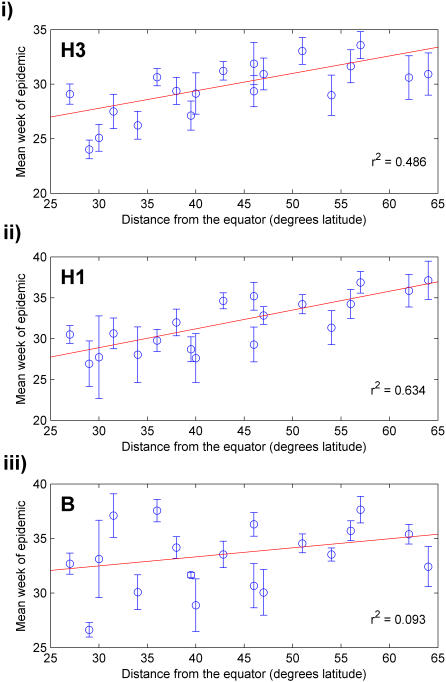
Mean type and subtype epidemic week versus distance from the equator. Mean week of each epidemic was determined for all countries, and the arithmetic mean (± SE) of these values for each country was plotted versus distance from the equator for (i) H3, (ii) H1, and (iii) B. The red lines indicate best fit lines for each type or subtype.

There was a positive correlation between increased distance from the equator, based on the absolute value of the latitude of the geographic center of the country, and later occurrence of epidemics for both H3 (regression between mean epidemic week and latitude, r^2^ = 0.49, *p*<0.001) and H1 (r^2^ = 0.63, *p*<0.001) (similar to [18]); however, no correlation was observed for B (r^2^ = 0.093, *p* = 0.10) (Figure 5). As a control, epidemic mean week was correlated against the longitude of the country's geographic center; no relationship was found for any type or subtype. Furthermore, latitude did not correlate with any other basic parameters for the studied countries, such as population size, total area, population density, or Gross Domestic Product (GDP).

We conducted a sensitivity analysis using the week of epidemic onset instead of the mean epidemic week, and found a more modest correlation with latitude for H3 (r^2^ = 0.39, *p* = 0.002) and H1 (r^2^ = 0.32, *p* = 0.006). In this case a modest positive relationship was also found for B (r^2^ = 0.37, *p* = 0.003). Of note, the week of onset is a more arbitrary indicator of epidemic timing than the mean week, and more prone to measurement error.

## Discussion

Through a quantitative analysis of laboratory-confirmed weekly type- and subtype-specific FluNet data from 19 countries, we found both support for previously-held seasonal and type and subtype dominance patterns [1], [2], [17], [19] as well as novel patterns of interhemispheric synchrony and latitudinal gradients in epidemic timing. First, the type and subtype data confirmed the well-established pattern of influenza epidemics occurring primarily during the winter months in temperate regions. Additionally, H3 appeared to be the most dominant subtype, followed by type B and then H1 [17]. No significant differences were found between the mean size of H1 and B epidemics; however, this outcome is most likely from B epidemics being more frequent but typically smaller than the H1 epidemics that did occur. Second, B epidemics occurred later in the season than H3 and H1 epidemics in the Northern Hemisphere, a pattern not previously described. Third, we found that influenza B was at times codominant with either H3 or H1, while these two subtypes rarely codominated with each other. This suggests that interference competition may be occurring between H3 and H1 and is consistent with a degree of genetic similarity between H3 and H1, as well as epidemiological studies suggesting cross-immunity between two subtypes [9], [10].

Despite strong seasonal patterns in flu epidemics, we found an extensive degree of temporal overlap of influenza activity in the Northern and Southern hemispheres, especially for H3 and B. Although the bulk of epidemics are confined to winter months, the background ‘noise’ of influenza activity during the interepidemic period may prove to have an impact on the dynamics of the subsequent epidemic, especially in the case of influenza B, for which local natural selection and persistence may be more significant than influenza A. These findings highlight the need for year-round viral surveillance and an increase in the number of countries that collect and report reliable incidence data at the subtype level.

Our analysis of interhemispheric synchrony indicated that H3 epidemics in the Northern and Southern hemispheres are not completely independent, even though they occur at distinct times of the year. The greater degree of interhemispheric synchrony we observed for H3 relative to H1 and type B, which was consistent with previous data on subtype epidemic synchrony in the U.S. [17], may be related to the same factors, such as its reproductive rate, that are also contributing to its greater observed dominance. This conclusion must be regarded with some degree of caution, however, as the sample size of countries studied between the two hemispheres is not comparable (15 countries in the Northern Hemisphere versus 4 countries in the Southern Hemisphere), and synchrony in H1 and type B may be observable if data over a longer time series were used. Furthermore, data for countries from tropical regions are needed to see if this synchrony between the Northern and Southern hemispheres is mediated by synchronous influenza activity in the Tropics, as would be expected if this region is acting as a reservoir, or source, of new viruses, as has been previously suggested [3], [18].

In an analysis of within-hemisphere type and subtype periodicity, we also found a preliminary hint of a biennial cycle (Figure 4), although this pattern was not significant at the p<0.05 level, in part because the data only cover a span of nine influenza seasons. Such a cycle could be dependent on a type or subtype's intrinsic period of oscillation, resulting from predictable patterns of immunity decay [20], [21]. However, an interacting factor is likely to be the punctuated antigenic changes that especially H3 and H1 undergo. Recent literature suggests that cluster transition years are associated with both increased incidence [22] and higher degrees of synchrony [17]; in the FluNet data analyzed here, we also see the occurrence of widespread epidemics among both H3 and H1 that seem to follow the emergence of new clusters. Future research, supported by data on antigenic types or genetic sequences as well as a longer historical perspective, could confirm the existence of periodicity in type and subtype dynamics and examine any spatiotemporal aspects of this pattern.

The most unanticipated finding of this study was the apparent positive relationship between latitude and epidemic timing for H3 and H1 (Figure 5). This relationship has a number of important implications. First, it supports the hypothesis that environmental factors have at least some impact on the geographic spread of influenza A, either through effects on transmission or host susceptibility [6], [19]. Second, this result suggests that this seasonal stimulus is consistent in both hemispheres, as the relative epidemic timing of influenza A was comparable in each [19]. Third, it suggests that influenza B is not regulated by this seasonal stimulus in the same manner as influenza A, a hypothesis that has not been previously stated in the literature.

Our analysis was focused on the apparent relationship between latitude and epidemic timing observed for type A but not type B influenza, where epidemic timing is measured by the mean epidemic week. We also tested the relationship between timing of epidemic onset and latitude, which gave more ambiguous results. However, it is recognized that estimates of the onset and end of an epidemic are less reliable measures of timing, because these estimates are based on very small numbers of cases and therefore more vulnerable to random fluctuations.

The observed relationship between epidemic timing and latitude is strengthened by several additional analyses we conducted (not shown). First, epidemic timing did not correlate with longitude, a control variable, making it less likely that our findings were purely the result of chance. Second, latitude was not correlated with any of the other parameters measured in the study (Table 1), reducing the likelihood of the relationship being the result of a confounder. Third, the observed lack of correlation between the timing of B epidemics and latitude cannot be explained by a lack of power as compared with A viruses, as there were more influenza B isolates than H1 recorded in the database.

Although the mechanisms behind this seasonal stimulus are still largely unresolved, prevailing hypotheses [4]–[6] suggest that epidemics should occur earlier the farther one moves away from the equator, as the winter season itself—and all of the factors associated with winter, such as indoor crowding, lower temperatures, decreased humidity, and reduced levels of direct sunlight—begins earlier in the year for these countries. Thus, this result suggests that annual epidemics of influenza A are not dominated by low level local circulation of the virus that can give rise to epidemics as specific environmental criteria are met. Rather, our results suggest that the annual epidemics are likely dominated by the introduction of new viruses from outside locations, a result that is consistent with the analysis of H3 phylogenetic patterns [23]. Future research could replicate phylogenetic studies using B virus sequences and test whether influenza B evolutionary dynamics are more dominated by local natural selection and persistence than H3.

Thus, it seems that countries far from the equator cannot in general experience epidemics of influenza A until after epidemics have begun in countries closer to the equator, from where the virus spreads northward or southward, depending on the hemisphere. These results therefore support the hypothesis that the tropics serve as an influenza reservoir in between influenza seasons in temperate regions [3], [18], at least for influenza A. In addition, this result is consistent with influenza epidemic patterns in Brazil, characterized by a combination of traveling waves originating from equatorial regions and seasonal conditions permissive to epidemic activity in higher latitude regions [18].

Our results highlight the importance of increasing year-round influenza surveillance at the type and subtype level. This need is especially pressing in tropical countries, where data collection is unfortunately just starting (in Asia or Latin America) or nonexistent (in Africa)—for instance, there were no tropical countries with consistent and reliable data available in the FluNet database. Systematic collection of data on viral activity and genetic sequences from tropical countries is essential to understanding the global circulation and evolutionary patterns of influenza and its types and subtypes.

In conclusion, much remains to be learned about the seasonal dynamics of human influenza; however, a picture is beginning to take shape of influenza A emerging annually in a wave from tropical to temperate regions and type and subtype dominance being governed by the interaction of antigenic changes with an intrinsic period of oscillation. Moreover, it seems clear that it is not safe to assume that the factors driving seasonality in influenza are necessarily the same across all types and subtypes; further experimental and epidemiological studies are needed to clarify any distinctions that may exist. Increasing the availability of viral surveillance data and extending the FluNet system are key to addressing these issues.

## Supporting Information

Movie S1 Movie showing time series of incidence data for H3. Maps show the natural log of adjusted weekly incidence data for H3. White indicates no data for a given week, and blue indicates a value of zero. The number of isolates increases on a log color scale, with purple indicating the maximum value for a given subtype. Plots below the map show the hemisphere weekly totals (blue = Southern Hemisphere, and red = Northern Hemisphere) and are given as a reference point for time as the movie progresses.
(1.84 MB MOV)

Movie S2 Movie showing time series of incidence data for H1. Maps show the natural log of adjusted weekly incidence data for H1. White indicates no data for a given week, and blue indicates a value of zero. The number of isolates increases on a log color scale, with purple indicating the maximum value for a given subtype. Plots below the map show the hemisphere weekly totals (blue = Southern Hemisphere, and red = Northern Hemisphere) and are given as a reference point for time as the movie progresses.
(1.57 MB MOV)

Movie S3 Movie showing time series of incidence data for B. Maps show the natural log of weekly incidence data for B. White indicates no data for a given week, and blue indicates a value of zero. The number of isolates increases on a log color scale, with purple indicating the maximum value for a given subtype. Plots below the map show the hemisphere weekly totals (blue = Southern Hemisphere, and red = Northern Hemisphere) and are given as a reference point for time as the movie progresses.
(1.87 MB MOV)

Figure S1 Mean annual incidence for each subtype. The percent of the total annual incidence belonging to a particular type or subtype for each season in each country was plotted on a color scale for (i) H3, (ii) H1, and (iii) B.
(9.54 MB TIF)
