# Supplementary figures and images for: Global Patterns in Seasonal Activity of Influenza A/H3N2, A/H1N1, and B from 1997 to 2005: Viral Coexistence and Latitudinal Gradients
Source: PLoS One. 2007 Dec 12;2(12):e1296. doi: 10.1371/journal.pone.0001296 (PMC2117904; doi:10.1371/journal.pone.0001296)

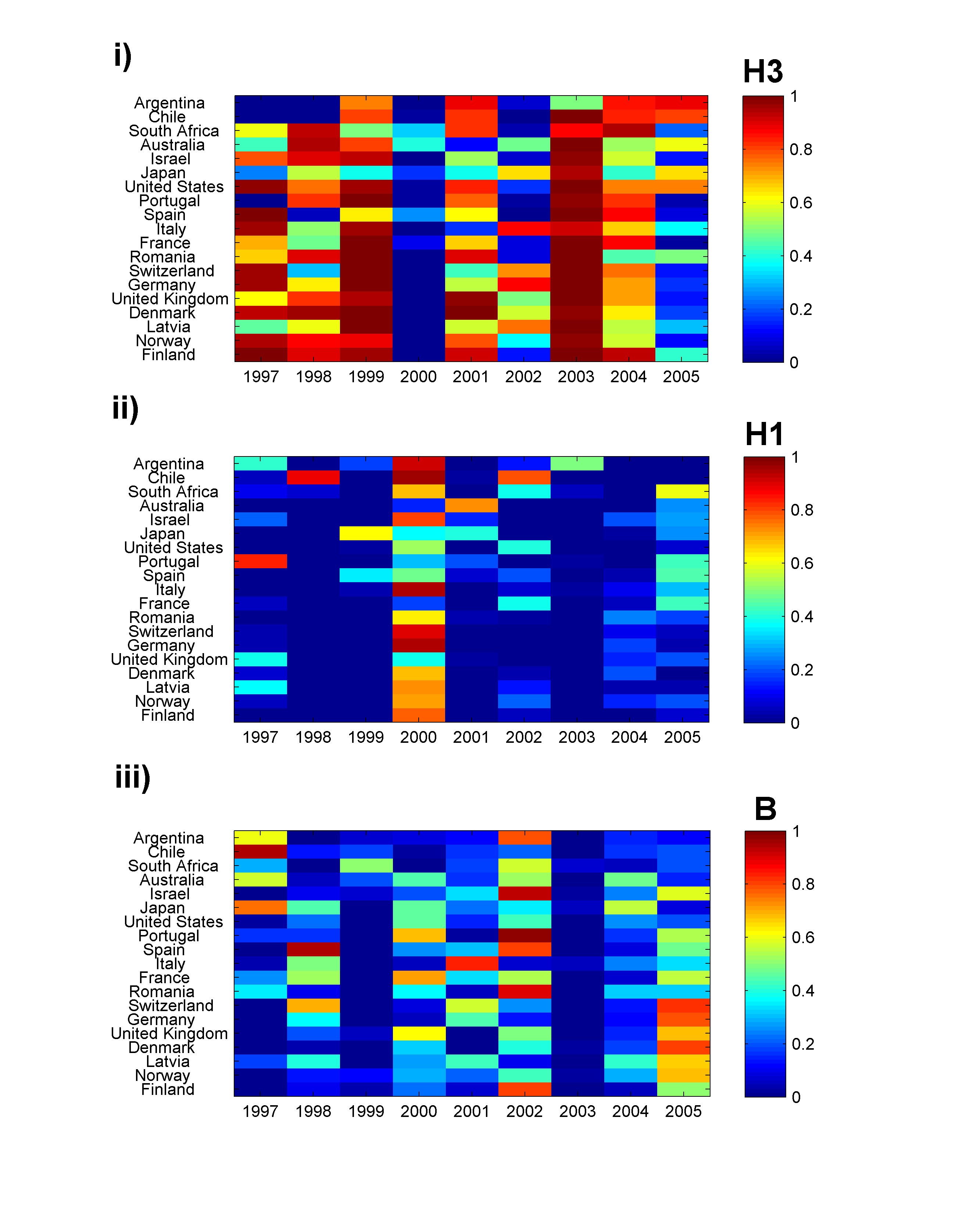

Supplement: Figure S1 — Mean annual incidence for each subtype. The percent of the total annual incidence belonging to a particular type or subtype for each season in each country was plotted on a color scale for (i) H3, (ii) H1, and (iii) B. (9.54 MB TIF) [file pone.0001296.s004.tif]
